# How to reduce the exposure risk of medical staff from SARS-CoV-2 by reducing environmental contamination: Experience from designated hospitals in China

**DOI:** 10.3389/fpubh.2022.963999

**Published:** 2022-11-29

**Authors:** Cui Zeng, Hengzhuo Liu, Yuling Jiang, Yuanyu Fu, Yuan Liu, Wei Chang, Tingting Li, Xun Huang, Chunhui Li

**Affiliations:** ^1^Teaching and Research Section of Clinical Nursing, Xiangya Hospital, Central South University, Changsha, China; ^2^Department of Infection Control, Xiangya Hospital, Central South University, Changsha, China; ^3^National Clinical Research Center for Geriatric Disorders, Xiangya Hospital, Central South University, Changsha, China; ^4^Xiangya Center for Evidence-Based Nursing Practice and Healthcare Innovation: A Joanna Briggs Institute (JBI) Affiliated Group, Xiangya Hospital, Central South University, Changsha, China; ^5^Department of Infection Control, Second Xiangya Hospital, Central South University, Changsha, China; ^6^Department of Infection Control, Hunan Provincial People's Hospital, Changsha, China; ^7^Department of Infection Control, The First People's Hospital of Huaihua City, Huaihua, China; ^8^Department of Infection Control, The First Affiliated Hospital of Shaoyang University, Shaoyang, China; ^9^Department of Infection Control, People's Hospital of Zhangjiajie, Zhangjiajie, China; ^10^Department of Infection Control, The First Affiliated Hospital of Hunan University of Traditional Chinese Medicine, Changsha, China

**Keywords:** SARS-CoV-2, environmental contamination, PPE contamination, designated hospitals for COVID-19, disease severity, close contacts, interventions

## Abstract

**Background:**

Using daily monitoring of environmental surfaces and personal protective equipment (PPE), we found an increase in environmental contamination since August 18, 2021, in a designated hospital for COVID-19 patients in China, which may lead to an increased risk of exposure to medical staff.

**Methods:**

To investigate the cause of increased environmental contamination and effect of our intervention, we obtained environmental samples at pre-intervention (August 18–21, 2021) and post-intervention (August 22–28, 2021) from six infection isolation rooms with windows for ventilation and other auxiliary areas at 105 and 129 sites before routine daily cleaning, respectively. In addition, we obtained PPE samples from 98 medical staff exiting the patient rooms/contaminated areas at 482 sites. Between August 22 and 24, 2021, we took measures to reduce environmental contamination based on sampling and inspection results.

**Findings:**

At pre-intervention, the positivity rates for contamination of environmental surfaces and PPE samples were significantly higher for critical patients (37.21 and 27.86%, respectively) than severely ill patients (25.00 and 12.50%, respectively) and moderately ill patients (0.00 and 0.00%, respectively) (Pearson's Chi-square: χ^2^ = 15.560, *p* = 0.000; Fisher's exact test: χ^2^ = 9.358, *p* = 0.007). Therefore, we inferred that the source of contamination of environmental surfaces and PPE was mainly the room of critically ill patients, likely through the hands of medical staff to the potentially contaminated areas. A critically ill patient had emergency tracheal intubation and rescue on August 18, 2021, due to worsened patient condition. The ventilator tube used for first aid did not match the ventilator, and the ventilator tube fell off multiple times on August 18–21, 2021, which may explain the increased contamination of environmental surfaces and PPE from critically ill patients, as well as lead to indirect contamination of potentially contaminated areas. The contamination positivity rates of environmental surfaces and PPE were reduced by replacing the appropriate ventilator catheter, limiting the number of people entering the isolation room simultaneously, increasing the frequency of environmental disinfection, standardizing the undressing process, setting up undressing monitoring posts to supervise the undressing process, and preventing the spread of virus infections in the hospital during an epidemic.

**Conclusions:**

Severe acute respiratory syndrome coronavirus 2 (SARS-CoV-2) was spread on object surfaces in isolation rooms mainly by touch, and the contamination of environmental surfaces and PPE was greater in rooms of patients with greater disease severity and higher surface touch frequency. Therefore, strict protective measures for medical staff, frequent environmental cleaning for isolation rooms, and compliance with mask wearing by patients when conditions permit should be advised to prevent SARS-CoV-2 spread in hospitals.

## Introduction

Severe acute respiratory syndrome coronavirus 2 (SARS-CoV-2) is sporadic in China and most often related to foreign transmission (1). However, cross-infection due to environmental contamination may also lead to infection transmission in designated hospitals (2). Information related to the environmental contamination of SARS-CoV-2 in coronavirus disease 2019 (COVID-19) wards and the exposure risk for close contacts of patients is critical for improving safety practices for medical staff and preventing SARS-CoV-2 transmission among the public (3). However, it is unknown whether individuals with different disease severities and different infection stages cause similar environmental pollution risk (4).

During daily environmental monitoring of a COVID-19 designated hospital in China, we found occasional contamination in contaminated areas and no contamination in potentially contaminated areas and cleaning areas. However, the contamination level was increased after August 18, 2021, which may lead to an increased risk of SARS-CoV-2 transmission in hospitals. Using frequent environmental sampling, we compared the contamination rate of environmental surfaces in different areas and personal protective equipment (PPE) of medical staff, and explored the reasons for worsened environmental contamination. The contamination rate was reduced through prevention and control measures. Until the end of the COVID-19 epidemic, virus infection did not spread in the hospital. In the present study, we describe the investigation process, results, and subsequent disposition of contamination of environmental surfaces and PPE.

## Methods

### Background

**Figure 1:**
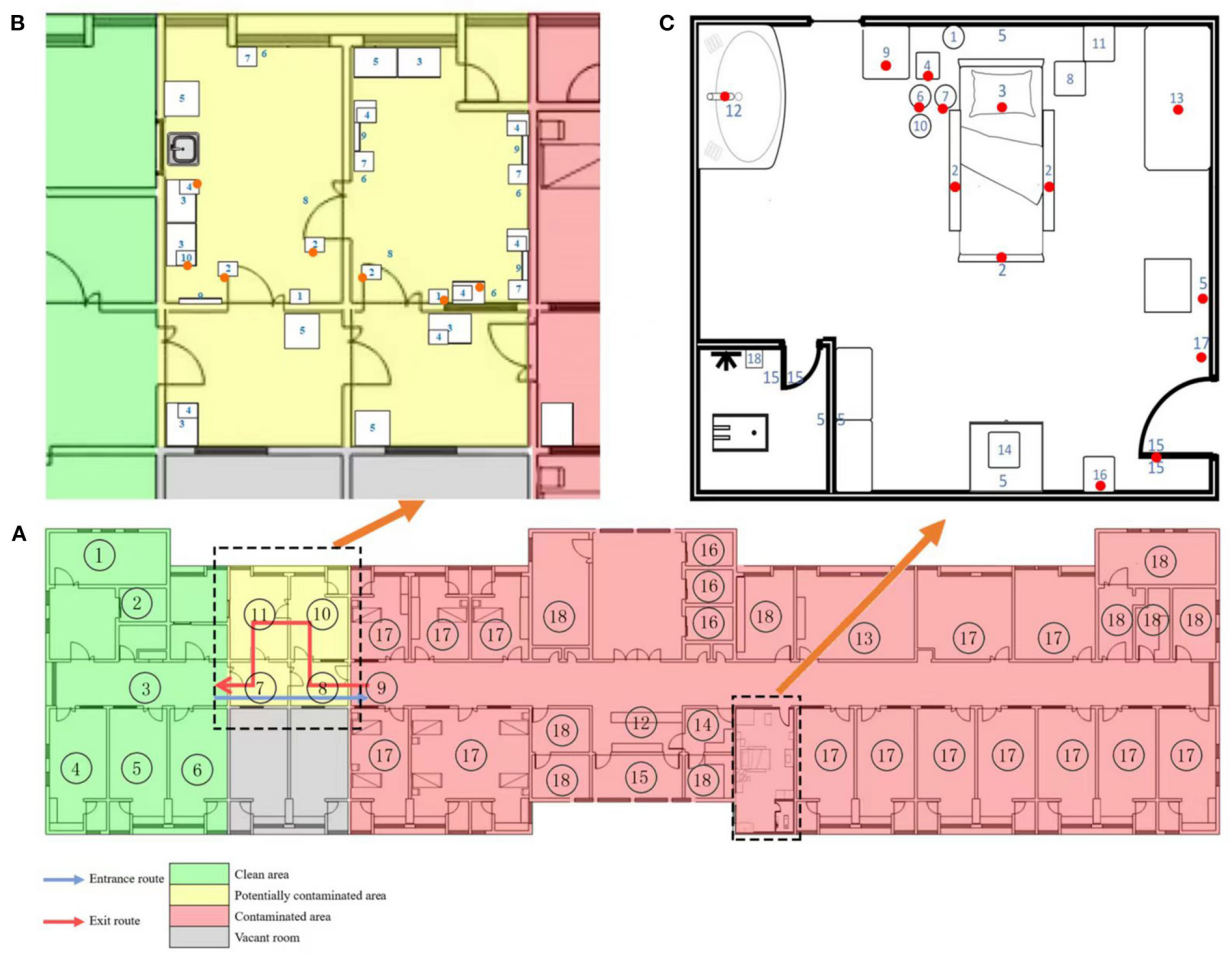
Layout of functional rooms in designated hospitals and sampling sites of critical patients' rooms and undressing rooms. **(A)** A general description of the layout of the inpatient wards where gray, green, yellow, and red regions represent the vacant rooms, cleaning areas, potentially contaminated areas, and contaminated areas, respectively. In field practice, entrances from cleaning areas to contaminated areas and exits from contaminated areas to cleaning areas were allocated on different floors. The road map for entry was: ① Stairs/② Cleaning elevator → ③ Clean aisle → ④ PPE dressing room/⑤⑥ Clean Storage → ⑦⑧ Buffer room → ⑨ Contaminated aisle. The road map for exit was: ⑨ Contaminated aisle → ⑧ Buffer room → ⑩ PPE undressing 1 → ⑪ PPE undressing 2 → ⑦ Buffer room → ③ Clean aisle → ④⑤⑥ shower room. The cleaning areas consist of PPE wearing room, shower room, conference room (which was not marked in this Figure, because it is on other clean floor), and cleaning elevator. The potentially contaminated areas include two undressing rooms, while the contaminated areas consist of patient rooms, ⑫ nurse station, ⑬ doctor's office, ⑭ dirty washing room, ⑮ treatment room, ⑯ contaminated elevator, ⑰ infected patients' rooms, and ⑱ other auxiliary area. **(B)** As shown in **(B)**, the potentially contaminated areas consist of two undressing rooms where PPE were removed stepwise in exit levels, along with two buffer rooms. The sampling number and sites include: 1. light switch, 2. door handle, 3. cabinet, 4. quick hand sanitizer press pump, 5. control panel of air disinfection machine, 6. wall above the medical waste barrel, 7. medical waste barrel, 8. entrance ground, 9. mirror, and 10. mask box. In undressing room 1, the face screen, goggles, and protective clothing were removed. One mixed sample swab tested positive for SARS-CoV-2, which covered the door handle, light switch, and cabinet surface. In undressing room 2 where masks were changed, one mixed sample tested positive, which was obtained from two door handles, a quick hand sanitizer press pump, and surface of a surgical mask box. The positive sites were marked with red and orange dots, red dots represent of single swab sampling positive and orange represent of mixed sampling positive. **(C)** Distribution of sample sites and positive sites in the critical patients' rooms is presented in **(C)**. Swabs were obtained from the corresponding sites of the following: 1. call bell attached to bed, 2. handrail of the bed, 3. bed surface, 4. ECG monitor, 5. Wall (only one swab from wall above the waste bin), 6. infusion pump, 7. enteral nutrition pump, 8. ventilator button, 9. surface of bedside table, 10. infusion stand, 11. blood gas analyzer button, 12. faucet, 13. medical staff desktop, 14. AED ready for use, 15. door handle (only one swab was obtained from the door handle leading to the corridor), 16. control panel of air disinfection machine, 17. light switch, and 18. ready-to-use commode. Positive sites are marked with red dots.

In August 2021, Zhangjiajie, China, reported an outbreak of the COVID-19 Delta variant that infected 76 individuals. According to disease severity, the patients were classified as critical (*n* = 1), severe (*n* = 2), moderate (*n* = 69), and asymptomatic (*n* = 4). All patients were admitted to the Zhangjiajie City Designated Hospital. In our hospital, isolated wards were set up in three areas (contaminated, potentially contaminated, and cleaning areas) and had two accesses (medical personnel and patient accesses). The layout of the functional rooms in our designated hospitals is shown in Figure 1. We conducted daily monitoring of environmental surfaces and PPE to investigate environmental virus contamination, and found that environmental contamination was increased after August 18, 2021. The contamination was significantly worse in the potentially contaminated areas, especially the undressing rooms, which previously had negative environmental monitoring. We focused our attention on the two dressing rooms in the designated hospitals in China: undressing room 1 was used to remove face screen, goggles, protective clothing, gloves, and boot covers, whereas undressing room 2 was used to remove hats and replace masks. Changing masks inside the contaminated room increases the risk of infection.

### Sampling

To determine the degree of environmental contamination and explore the reasons underlying the worsened contamination, we increased the sampling points and number of samples. Between August 18–21, 2021 (pre-intervention) and August 22–28, 2021 (post-intervention), environmental samples were obtained from six isolation rooms with windows for ventilation (1, 2, and 3 single rooms for critical, severely ill, and moderately ill patients, respectively) and other auxiliary areas from 105 and 129 sites, respectively, before routine daily cleaning in a COVID-19 designated hospital in China. PPE samples were obtained from 98 medical staff at 482 exit sites from the patient rooms/contaminated areas. The distribution of samples in rooms of critical patients is described in Figure 1. Sampling of protective equipment for medical personnel was completed before they left the isolation ward and sampling sites include back of the head part of protective clothing, surface of front of boot cover, front surface of face screen, goggles, upper front part of protective clothing, sleeves of protective clothing.

Sterile premoistened swabs were used to collect the samples, which were immediately stored at 4°C in the hospital prior to transfer to a biosafety shelter laboratory-2 (BSL-2). We tested surface samples for open reading frame (ORF) 1ab, nucleoprotein (N) genes, and envelope protein (E) genes of SARS-CoV-2 by quantitative real-time polymerase chain reaction (PCR). A sample was considered positive if any of the three targets (ORF 1ab, N, and E) demonstrated apparent logarithmic phase in the amplification curve and a cycle threshold (Ct) value < 200.

### Data collection

Clinical characteristics, including symptoms, disease course, Ct values, supplemental oxygen requirement, nebulization therapy, and ventilator-assisted breathing, were recorded. Disease severity was recorded according to the Diagnosis and Treatment Protocol for Novel Coronavirus Pneumonia (Trial Version 8) (5).

### Interventions

Interventions were developed based on the problems that could be related to contamination identified by the inspectors, including using an appropriate ventilator tube, standardizing the undressing process, setting monitoring posts to supervise the undressing process, increasing the frequency of environmental disinfection of severe and critical patient rooms, and limiting the number of people who enter the isolation room at the same time (< 4 people). The upper limit on the number of people allowed in was mainly based on the size of the room, and this restriction was only for routine work, does not include rescue and other special circumstances.

Before the intervention, the potentially contaminated areas and cleaning areas were cleaned by medical staff one to four times a day using Clinell universal wipes. The floor was cleaned twice daily using a disinfectant with an effective chlorine concentration of 1,000 mg/L. High-touch surfaces (e.g., call bell attached to the bed, handrail, bedside table, and monitor) were cleaned twice daily in the room of moderately ill patients using Clinell universal wipes. Rooms for severely and critically ill patients were cleaned by medical staff four times a day using Clinell universal wipes before and after the intervention, as well as in special circumstances, such as rescue and ventilator tube detachment.

### Statistical analysis

SPSS software (version 20) was used for data analyses. Percentage positivity was calculated for items in rooms of patients with different disease severity, rooms in other auxiliary areas, and different components of PPE from medical staff at different positions. Mann–Whitney *U* test and Kruskal–Wallis test were used to compare the Ct values between patients with different disease severities and between patients with and without environmental surface contamination. Differences in the positivity rates of SARS-CoV-2 before and after intervention and between different hospital areas, medical items, and PPE were assessed using Chi-Square tests or Fisher's exact test. Two-tailed tests were used, and *p*-values < 0.05 were considered statistically significant.

## Results

### Characteristics of patients

Six patients were enrolled in the study, including one critical patient, two severely ill patients, and three moderately ill patients. All patients experienced COVID-19 symptoms. The latest Ct values from clinical samples before environmental sampling are shown in Table 1. During the isolation period, patients wore masks, except for dining, drinking, personal hygiene, oxygen inhalation, or mechanical ventilation. Median Ct values of the clinical specimens for patients with and without environmental surface contamination were as follows: N: 28.3, 1ab: 30.57, E: 26.69 [interquartile range (IQR), N: 23.39–31.39, 1ab: 25.91–37.24, E: 24.06–28.03] and N: 31.32, 1ab: 33.83, E: 26.4 (IQR, N: 24.03–36.03, 1ab: 27.16–37.28, E: 24.10–27.86), respectively. The median days of illness among patients with and without environmental surface contamination were 17 (IQR, 15–20) and 21.5 (IQR, 13–25) days, respectively.

**Table 1:** Characteristics of patients.

**Disease severity**	**Patient**	**Age**	**Gender**	**Vaccine**	**Supplemental oxygen requirement/nebulization therapy/ventilator-assisted breathing**	**Symptoms**	**Days of illness when samples were collected**	**Cycle threshold value from clinical samples**	**Results of environmental**
Critical	A	57	Female	No	Ventilator-assisted breathing and oxygen inhalation	Coma and sedation	15	N: 29.05 1ab: 30.57 E: 28.03	+
							16	N: 24.38 1ab: 25.91 E: 26.69	+
							17	N: 23.39 1ab: 26.48 E: 24.45	+
							21	N: 25.21 1ab: 28.59 E: 24.89	–
							22	N: 24.03 1ab: 27.16 E: 24.16	–
							23	N: 32.43 1ab: 35.42 E: 25.99	–
Severe	B	60	Female	No	Non-invasive ventilator-assisted breathing and oxygen inhalation	Diarrhea	20	N: 28.30 1ab: 30.67 E: 24.06	+
					Oxygen inhalation	Cough, sputum production	25	N: 30.21 1ab: 32.25 E: 26.80	–
	C	40	Male	No	Oxygen inhalation	Cough, sputum production	18	N: 31.39 1ab: 37.24 E: 27.80	+
						None	23	N: 34.03 1ab: 37.87 E: 27.86	–
Moderate	D	55	Female	Yes	Oxygen inhalation	Diarrhea	13	N: 35.51 1ab: 37.24 E: 27.80	–
	E	38	Male	Yes	Oxygen inhalation	Cough, fever	19	N: 36.03 1ab: 37.28 E: 27.86	–
	F	53	Female	Yes	Oxygen inhalation	None	19	N: 29.06 1ab: 30.19 E: 24.10	–

^A^Moderate cases showed fever and respiratory symptoms with radiological findings of pneumonia. Adult severe cases fulfilled any of the following criteria: (1) respiratory distress (≥30 breaths/min); (2) oxygen saturation ≤ 93% at rest; and (3) arterial partial pressure of oxygen (PaO_2_)/fraction of inspired oxygen (FiO_2_) ≤ 300 mmHg. (4) Cases with chest imaging that showed obvious lesion progression of >50% within 24–48 h were managed as severe cases. Childhood severe cases fulfilled any of the following criteria: (1) high-grade fever that lasts for more than 3 days; (2) tachypnea [RR ≥ 60 breaths/min (BPM) for infants aged below 2 months; RR ≥ 50 BPM for infants aged 2–12 months; RR ≥ 40 BPM for children aged 1–5 years; and RR ≥ 30 BPM for children above 5 years] independent of fever and crying; (3) oxygen saturation ≤ 92% on finger pulse oximetry recorded at rest; (4) labored breathing (moaning, nasal fluttering, and infrasternal, supraclavicular, and intercostal retraction), cyanosis, and intermittent apnea; (5) lethargy and convulsions; and (6) difficulty in feeding and signs of dehydration. Critical cases fulfilled any of the following criteria: (1) respiratory failure and need of mechanical ventilation; (2) shock; and (3) organ failure requiring ICU care.

^B^Clinical samples included nasopharyngeal or oropharyngeal swabs. The most recent results prior to the environmental sampling were recorded. Ct refers to the number of cycles required for the fluorescent signal to cross the threshold during reverse transcriptase–polymerase chain reaction; a lower cycle threshold value indicates a higher viral load.

^C^If there was one positive result in the environmental samples, it was marked as +.

^D^The Ct values of clinical specimens from patients with or without environmental surface contamination showed no statistical difference (Mann–Whitney U test, N: Z = −1.610, p = 0.107, 1ab: Z = −1.246, p = 0.213, E: Z = −0.073, p = 0.941).

^E^The Ct values of clinical specimens from patients with different disease severity showed no statistical difference (Kruskal–Wallis test, N: H = 5.885, p = 0.053, 1ab: H = 5.938, p = 0.051, E: H = 0.331, p = 0.847).

^*F*^The days of illness when samples were collected for patients with and without environmental surface contamination showed no statistical difference (Mann–Whitney U test, Z = −1.908, p = 0.056).

### Environmental sampling

The percentage positivity of contaminated, potentially contaminated, and clean areas before intervention were 26.03% (19/73), 6.06% (2/33), and 0.00% (0/36), respectively (Tables 2, 3), with significant differences between the areas (Pearson's Chi-square: χ^2^ = 15.560, *p* = 0.000).

**Table 2:** Results of SARS-CoV-2 testing in infection isolation rooms before the intervention.

**Sites**	**No. of positive samples/no. of total samples (positivity rate, %)**		
	**Critically ill patients' rooms**	**Severely ill patients' rooms**	**Moderately ill patients' rooms**	**Total**
Call bell attached to bed	0/1 (0.00)	0/2 (0.00)	0/3 (0.00)	0/6(0.00)
Handrail	4/9 (44.44)	1/4 (25.00)	0/4 (0.00)	5/17(29.41)
Bedside table	1/2 (50.00)	2/2 (100.00)	0/3 (0.00)	3/7(42.86)
Wall	1/7 (14.29)	0/2 (0.00)	0/3 (0.00)	1/12(8.33)
Monitor	1/3 (33.33)	0/2 (0.00)	–	1/5(20.00)
Infusion pump	1/2 (50.00)	–	–	1/2 (50.00)
Ventilator button	0/2 (0.00)	–	–	0/2 (0.00)
Door handle	2/5 (40.00)	–	–	2/5 (40.00)
Bed surface	1/1 (100.00)	–	–	1/1 (100.00)
Ready-to-use commode	0/1 (0.00)	–	–	0/1 (0.00)
Medical staff desktop	1/1 (100.00)	–	–	1/1 (100.00)
Faucet	1/1 (100.00)	–	–	1/1 (100.00)
AED ready for use	0/1 (0.00)	–	–	0/1 (0.00)
Button of blood gas analyzer	0/1 (0.00)	–	–	0/1 (0.00)
Enteral nutrition pump	1/2 (50.00)	–	–	1/2 (50.00)
Control panel of air disinfection machine	1/2 (50.00)	–	–	1/2 (50.00)
Light switch	1/1 (100.00)	–	–	1/1 (100.00)
Infusion stand	0/1 (0.00)	–	–	0/1 (0.00)
Total	16/43 (37.21)	3/12 (25.00)	0/13 (0.00)	19/68 (27.94)

^A^One swab was taken from each site, and samples were collected before routine cleaning.

^B^Samples from the walls of severely and moderately ill patients' rooms were obtained from the head end walls. The walls of critically ill patients' rooms include the walls of the head and foot ends, bathroom wall, above the clean shelf, above the medical waste barrel, and in the bathroom. Only the wall above the medical waste barrel showed a positive result.

^C^The samples from handrails of severely and moderately ill patients' rooms were obtained from the left and right sides of the beds. The handrails in critical patients' rooms include the handrails on left, right, and foot ends of the bed. However, only the samples from the foot end of the bed showed negative results.

^D^The red cell indicates a positive nucleic acid test, and the green cell indicates a negative nucleic acid test.

**Table 3:** Results of SARS-CoV-2 testing from other auxiliary areas before intervention.

**Other auxiliary areas**		**No. of positive samples/no. of total samples (positivity rate, %)**	**Sites**
Contaminated areas	Nurse station	0/3 (0.00)	Air outlet and return panel of air disinfection machine, computer mice, and keyboard
	Doctor's office	0/2 (0.00)	Desktop, work phone, pen, door handle, computer mice, and keyboard
Total		0/5(0.00)	
Potentially contaminated areas	Buffer room	0/9 (0.00)	Door handle, light switch, control panel of air disinfection machine, quick hand sanitizer press pump, cabinet, cover of the medical waste barrel, and wall above the medical waste barrel
	Undressing room 1	1/7 (14.29)	Light switch, door handle, cabinet, quick hand sanitizer press pump, control panel of air disinfection machine, wall above the medical waste barrel, and entrance ground
	Undressing room 2	1/5 (20.00)	Door handle, control panel of air disinfection machine, light switch, cabinet, quick hand sanitizer press pump, mask box, wall above the medical waste barrel, and entrance ground
	Dressing room 1	0/12 (0.00)	Door handle, light switch, control panel of air disinfection machine, quick hand sanitizer press pump, desktop, cover of the medical waste barrel, and wall above the medical waste barrel
Total		2/33(6.06)	
Cleaning areas	CT operating room	0/1 (0.00)	Walkie-talkie, computer mice and keyboard, doorbell button, control panel of air disinfection machine, and cover of the medical waste barrel
	Cleaning area of clinical laboratory department	0/12 (0.00)	Door handle, stair handrail, duty room bed rails, water dispenser switch, access control button, light switch, meeting table, air conditioner button, and chair
	Dressing room	0/12 (0.00)	Door handle, quick hand sanitizer press pump, washing machine, control panel of air disinfection machine, cover of the medical waste barrel, shoe shelf, entrance ground, and faucet
	Elevator	0/2 (0.00)	Button
	Multidisciplinary team meeting room	0/5 (0.00)	Computer mice and keyboard, desktop, seat, control panel of air disinfection machine, door handle, and printer
	Medical team commuter car	0/4 (0.00)	Handrail and chair
Total		0/36(0.00)	

^A^One swab was obtained from each item for all sites and samples were collected before routine cleaning.

^B^The red cell indicates a positive nucleic acid test, and the green cell indicates a negative nucleic acid test.

The positivity rate was significantly higher for rooms of critical patients (16/43, 37.21%) than of severely ill patients (3/12, 25.00%) and moderately ill patients (0/13, 0.00%). Fixed items (call bell attached to bed, handrail, bedside table, and wall) showed different positivity rates between rooms of patients with different disease severities. The highest rates were observed for bedside tables (42.86%, 3/7), followed by handrails (29.41%, 5/17), walls (8.33%, 1/12), and call bells attached to the bed (0.00%, 0/6).

The distribution of positive samples in rooms of critical patients was not significantly related to the distance from patients' mouths and noses but was more likely to be observed in surfaces frequently touched by medical staff.

### PPE sampling

The pre-intervention PPE sampling results for staff at different positions with different job content are described in Table 4. PPE from staff involved in medical waste packaging and transfer, psychological counseling, environmental sampling, patient sampling, physical examination, environmental cleaning and disinfection, equipment maintenance, and care of moderate patients tested negative for environmental contamination. The highest contamination rate of PPE samples from medical staff involved in care of critical patients was from samples collected from the upper front surface of shoes (38.46%, 10/26), followed by upper front part of protective clothing (36.67%, 11/30), sleeves of protective clothing (36.67%, 11/30), back of the head cover of protective clothing (15.38%, 4/26), and goggles (0.00%, 0/6). The percentage positivity of PPE samples from medical staff involved in the care of moderate patients (0.00%, 0/15) and severe patients (12.50%, 5/40) were lower than that for staff involved in care of critical patients (27.86%, 39/140) (Fisher's exact test: χ^2^ = 9.358, *p* = 0.007).

**Table 4:** Results of SARS-CoV-2 testing for PPE from medical staff exiting the contaminated areas before intervention.

**Medical staff**		**No. of positive samples/no. of total samples (positivity rate, %)**						**Total**
		**Back of the head part of protective clothing**	**Surface of front of boot cover**	**Front surface of face screen**	**Goggles**	**Upper front part of protective clothing**	**Sleeves of protective clothing**	
No close contact with the patient	Medical waste packaging and transfer	0/6 (0.00)	0/6 (0.00)	0/5 (0.00)	0/1 (0.00)	0/6 (0.00)	0/6 (0.00)	0/30 (0.00)
	Environmental sampling	0/5 (0.00)	0/5 (0.00)	0/5 (0.00)	–	0/5 (0.00)	0/5 (0.00)	0/25 (0.00)
	Psychological counseling	0/4 (0.00)	0/4 (0.00)	0/3 (0.00)	0/1 (0.00)	0/4 (0.00)	0/4 (0.00)	0/20 (0.00)
	Environmental cleaning and disinfection	0/1 (0.00)	0/1 (0.00)	0/1 (0.00)	–	0/1 (0.00)	0/1 (0.00)	0/5 (0.00)
	Equipment maintenance	0/1 (0.00)	0/1 (0.00)	–	0/1 (0.00)	0/1 (0.00)	0/1 (0.00)	0/5 (0.00)
	Total	0/13 (0.00)	0/13 (0.00)	0/11 (0.00)	0/2 (0.00)	0/13 (0.00)	0/13 (0.00)	0/65 (0.00)
Close patient contact	Patient sampling	0/1 (0.00)	0/1 (0.00)	–	0/1 (0.00)	0/1 (0.00)	0/1 (0.00)	0/5 (0.00)
	Physical examination	0/1 (0.00)	0/1 (0.00)	–	0/1 (0.00)	0/1 (0.00)	0/1 (0.00)	0/5 (0.00)
	Psychological counseling	0/4 (0.00)	0/4 (0.00)	0/3 (0.00)	0/1 (0.00)	0/4 (0.00)	0/4 (0.00)	0/4 (0.00)
	Medical staff of critical patients	4/26 (15.38)	10/26 (38.46)	2/17 (11.76)	1/11 (9.09)	11/30 (36.67)	11/30 (36.67)	39/140 (27.86)
	Medical staff of severely ill patients	1/8 (12.50)	0/8 (0.00)	0/3 (0.00)	0/5 (0.00)	2/8 (25.00)	2/8 (25.00)	5/40 (12.50)
	Medical staff of moderately ill patients	0/3 (0.00)	0/3 (0.00)	0/2 (0.00)	0/1 (0.00)	0/3 (0.00)	0/3 (0.00)	0/15 (0.00)
	Total	5/43 (11.63)	10/43 (23.26)	2/25 (0.08)	1/20 (5.00)	13/47 (27.66)	13/47 (27.66)	44/225 (19.56)
Total		5/56 (8.93)	10/56 (17.86)	2/36 (1.06)	1/22 (4.55)	13/60 (21.67)	13/60 (21.67)	44/290 (15.17)

^A^One swab was obtained from each site.

^B^Face screen and goggles were not worn simultaneously.

^C^The percentage positivity of PPE from medical staff with and without close contact showed no significant difference (Yates' correction Chi-square: χ^2^ = 3.105, p = 0.078).

^D^The red cell indicates a positive nucleic acid test, and the green cell indicates a negative nucleic acid test.

### Cause of worsened contamination

According to the sampling results, contamination was mainly concentrated in the rooms of critically ill patients and PPE of medical staff caring for critically ill patients. The contamination was caused by the hands of medical staff in potentially contaminated areas. To investigate the reason for the aggravation of environmental contamination in rooms of critically ill patients, we reviewed the treatment activities during August 18–21, 2021. We found that a critically ill patient underwent emergency tracheal intubation and rescue on August 18, 2021, due to worsening condition. However, the ventilator tube used during first aid was not appropriate for the ventilator, and the ventilator tube fell off multiple times on August 18–21, 2021. This may have led to viral contamination of environmental surfaces. In addition, the number of medical staff entering the patients' rooms and unnecessary contact were increased due to emergency operations, such as tracheal intubation, rescue, and de-intubation. Furthermore, the contamination can be carried to other areas by the hands of medical staff.

### Sampling results before and after intervention

The results of sampling investigations before and after the intervention are shown in Table 5. After interventions, the contamination positivity rates of PPE and environmental surfaces in the contaminated areas were significantly decreased.

**Table 5:** Results of sampling investigation before and after intervention.

**No. of positive samples/no. of total samples (positivity rate, %)**					
**Date**		**PPE**	**Surfaces of contaminated areas**	**Surfaces of potentially contaminated areas**	**Surfaces of cleaning areas**
Before intervention	August 18	3/92 (3.26%)	2/4 (50.00%)	0/8 (0.00%)	0/8 (0.00%)
	August 19	7/95 (7.37%)	2/22 (9.09%)	0/8 (0.00%)	0/8 (0.00%)
	August 20	18/50 (36.00%)	5/27 (18.52%)	1/8 (12.50%)	0/8 (0.00%)
	August 21	16/53 (30.19%)	10/20 (50.00%)	1/9 (11.11%)	0/8 (0.00%)
	Total	44/290 (15.17%)	19/73 (26.03%)	2/33 (6.06%)	0/32 (0.00%)
After intervention	August 25	1/42 (2.38%)	0/13 (0.00%)	0/8 (0.00%)	0/8 (0.00%)
	August 26	0/50 (0.00%)	0/2 (0.00%)	0/8 (0.00%)	0/8 (0.00%)
	August 27	0/50 (0.00%)	0/7 (0.00%)	0/8 (0.00%)	0/8 (0.00%)
	August 28	0/50 (0.00%)	0/10 (0.00%)	0/8 (0.00%)	0/8 (0.00%)
	Total	1/192 (0.52%)	0/32 (0.00%)	0/32 (0.00%)	0/32 (0.00%)
χ^2^		29.297	10.169	2.773	–
*P*		0.000	0.001	0.492	–

^A^The red cell indicates a positive nucleic acid test, and the green cell indicates a negative nucleic acid test.

## Discussion

We found significant contamination of surfaces surrounding the critical patients and PPE from medical staff involved in the care of critically ill patients. Sporadic positive results were obtained from potentially contaminated areas and rooms of severely ill patients. The cleaning areas showed no contamination due to routine disinfection and correct use of PPE, as observed during the daily monitoring. We believe that the contamination of environmental surfaces around the critically ill patients may be related to the lack of a tight seal of the ventilator tube due to mismatch between the tube and its ventilator, which led to several episodes of ventilator tube detachment. In addition, critically ill patients often have a greater number of caregivers than other patients, which leads to a greater number of people staying in a single room simultaneously and an increased cumulative number of people entering the room, which can reach up to 10 and 20, respectively. A high number of people entering the room inevitably lead to increased unnecessary contact, which leads to environmental contamination. We also observed occasional contamination in potentially contaminated areas (undressing rooms 1 and 2) when the contaminated areas and PPE were heavily contaminated. In undressing room 1, the staff were asked to remove the protective clothing, gloves, and boot covers. In addition, they would remove the hat and wear a new mask in undressing room 2. Therefore, the infection risk would be increased in case of contamination in the undressing rooms. The sampling results showed improvement with measures such as using an appropriate ventilator tube, standardizing the undressing process, and limiting the number of people who enter the isolation room simultaneously. The positive contamination rates of PPE and environmental surfaces in the contaminated areas were significantly decreased, and no staff members at our designated hospital were infected with SARS-CoV-2 until the end of the outbreak, indicating that appropriate precautions could effectively prevent infection.

Despite the small sample, our study showed the correlation of surface contamination with different disease severities at the individual patient level. The contamination level of patient rooms before the intervention correlated with the disease severity of patients. Rooms with patients with severe diseases were more contaminated. However, the median Ct values of the clinical specimens from patients with different disease severities showed no significant differences (critical patients, N: 34.8, 1ab: 37.88, E: 25.44; severely ill patients, N: 30.8, 1ab: 34.75, E: 27.3; and moderately ill patients, N: 35.51, 1ab: 37.24, E: 27.8).

The main transmission routes for SARS-CoV-2 are respiratory droplets and close contact (6). In rooms of critical patients, environmental contamination could be attributed to direct touch contamination by healthcare workers after contact with infected respiratory fluids. Negative results of items disinfected for use (automated external defibrillator (AED), washroom materials, and call bell) that were located close to the patient support this hypothesis. Transmission by respiratory droplets was not observed in our study, which may be related to the use of surgical masks by patients and tracheal intubation in the wards. Previous studies suggest that healthcare workers may not be infected after exposure to confirmed COVID-19 patients despite not using airborne precautions (7). We obtained samples from the walls of rooms of critical patients at different distances from the patients' nose and mouth, including the wall at the head and foot ends of patients, bathroom, above the clean shelf, and above the medical waste barrel. Only the wall above the medical waste barrel was contaminated, which may be related to aerosol generated during disposal of medical waste. The surfaces that were frequently touched by medical staff or patients showed relatively higher positivity rates, suggesting that medical staff should follow hand hygiene practices immediately after patient contact.

Previous studies suggested that surface sampling showed that PCR-positivity at high-touch surfaces was associated with nasopharyngeal viral loads and peaked at approximately day 4–5 of symptoms (8–11). However, in our study, extent of environmental contamination did not correlate with disease course or viral loads.

The positive PPE samples were unsurprising because of the close contact with patients. We sampled the sites that have not been previously studied extensively, instead of soles and gloves, which are known to be polluted and be affected by daily disinfection (12–15). In previous studies, the front surface of face screen and goggles was contaminated by infected patients' respiratory droplets or infectious aerosols (16); however, in our study, the percentage positivity was lower than expected (0.08 and 5.00%, respectively), which supports the hypothesis that contact is the main mode of transmission of SARS-CoV-2 in our wards where patients have ideal compliance with wearing masks, which is consistent with the environmental sampling results. The contamination of the back of the head component of protective clothing (8.93%, 5/56) cannot be explained but may be related to certain habitual movements of the medical staff. The highest positivity rate was detected from the upper front part (21.67%, 13/60) and sleeves (21.67%, 13/60) of the protective clothing. Contamination was mainly concentrated on the PPE of caregivers of critical and severely ill patients, consistent with environmental pollution. However, no significant difference was observed in the percentage of positive samples across the PPE samples obtained from medical staff with and without close contact with patients.

## Conclusions

SARS-CoV-2 was distributed on the object surfaces in isolation rooms mainly due to touch, and the contamination of the environment and PPE was greater in rooms of patients with greater disease severity and frequent surface touching. Given the potentially higher exposure risk for medical staff in close contact with severely ill patients, strict protective measures should be taken for medical staff working in the rooms of critically ill patients to prevent the spread of SARS-CoV-2 in hospitals.

## Limitations

This study had several limitations. First, this study was performed in a designated hospital during an outbreak. Second, the study methodology was inconsistent, and the sample size was small; therefore, the results might not reflect the conditions in hospitals that are operating at full capacity. Third, the air particle size distribution of SARS-CoV-2 was not determined and should be evaluated in future studies to demonstrate the airborne transmission potential of SARS-CoV-2. Finally, viral culture was not performed to demonstrate the viability of SARS-CoV-2.

## Data availability statement

The original contributions presented in the study are included in the article/Supplementary material, further inquiries can be directed to the corresponding author/s.

## Ethics statement

Informed consent was waived as clinical data were collected as part of outbreak investigation.

## Author contributions

CZ was a major contributor in writing the manuscript. CL made contributions to conception and design. CZ, HL, YJ, YF, YL, WC, TL, and XH made substantial contributions to acquisition of data and interpretation of data. All authors read and approved the final manuscript.

## Funding

This work was supported by the National Key Research and Development Program of China Research on the Precision Diagnosis, Treatment, and Integrated Prevention, Control for the elderly with common infectious disease (no. 2020YFC2005403) and the Key Research and Development Projects of Hunan Province (nos. 2020SK3027 and 2020SK3028).

## Conflict of interest

The authors declare that the research was conducted in the absence of any commercial or financial relationships that could be construed as a potential conflict of interest.

## Publisher's note

All claims expressed in this article are solely those of the authors and do not necessarily represent those of their affiliated organizations, or those of the publisher, the editors and the reviewers. Any product that may be evaluated in this article, or claim that may be made by its manufacturer, is not guaranteed or endorsed by the publisher.

## Abbreviations

SARS-CoV-2: Severe acute respiratory syndrome coronavirus 2
COVID-19: Coronavirus disease 2019
PPE: Personal protective equipment
BSL-2: Biosafety shelter laboratory-2
ORF: Open reading frame
N: Nucleoprotein genes
E: Envelope protein genes
PCR: Polymerase chain reaction
Ct: Cycle threshold
IQR: Interquartile range
PaO_2_: Arterial partial pressure of oxygen
FiO_2_: Fraction of inspired oxygen
RR: Respiration rate
BPM: Beats per minute
ICU: Intensive care unit
AED: Automated external defibrillator
CT: Computed tomography.
