# Supplementary material for: How to reduce the exposure risk of medical staff from SARS-CoV-2 by reducing environmental contamination: Experience from designated hospitals in China
Source: Front Public Health. 2022 Nov 29;10:963999. doi: 10.3389/fpubh.2022.963999 (PMC9745115; doi:10.3389/fpubh.2022.963999)
Supplement: Supplementary file 1 [file Table_1.DOCX]

***Interventions***

Interventions were developed based on the problems that could be related to contamination identified by the inspectors, including using an appropriate ventilator tube, standardizing the undressing process, setting monitoring posts to supervise the undressing process, increasing the frequency of environmental disinfection of severe and critical patient rooms, and limiting the number of people who enter the isolation room at the same time(<4 people).

1.Using an appropriate ventilator tube

The ventilator tube used for first aid did not match the ventilator, and the ventilator tube fell off multiple times on August 18–21, 2021, which may explain the increased contamination of environmental surfaces and PPE from critically ill patients, as well as lead to indirect contamination of potentially contaminated areas. So we need to change an appropriate ventilator tube.

2.Standardizing the undressing process, setting monitoring posts to supervise the undressing process

Removing protective equipment should be done more carefully than putting it on, which is more likely to spread infection. In the environmental sampling before intervention, we found that environmental contamination occurred in both undressing room 1 and undressing room 2. In China, undressing room 1 was used to remove face screen, goggles, protective clothing, gloves, and boot covers, whereas undressing room 2 was used to remove hats and replace masks. Therefore, the infection risk would be increased in case of contamination in the undressing rooms. We thought the contamination was caused by improper removal of PPE or lax hand hygiene.

We organized training about precautions for wearing and taking off PPE for all personnel entering the isolation ward. In particular, we developed detailed procedures for removing protective equipment, and required each team member to learn and pass the assessment. We also set up a 24h supervision post in the undressing room 1 to observe the situation of each team member taking off PPE, and timely disinfect the contaminated environmental surface during the removal of PPEt, and timely clean up the medical waste in the undressing room 1and 2 to avoid the environmental contamination caused by the full of medical waste.

3.Increasing the frequency of environmental disinfection of severe and critical patient rooms

Before the intervention, the potentially contaminated areas and cleaning areas were cleaned by medical staff one to four times a day using Clinell universal wipes. The floor was cleaned twice daily using a disinfectant with an effective chlorine concentration of 1000 mg/L. High-touch surfaces(e.g., call bell attached to the bed, handrail, bedside table, and monitor) were cleaned twice daily in the room of moderately ill patients using Clinell universal wipes. Rooms for severely and critically ill patients were cleaned by medical staff four times a day using Clinell universal wipes before and after the intervention, as well as in special circumstances, such as rescue and ventilator tube detachment.

4.Limiting the number of people who enter the isolation room at the same time(<4 people)

We limited the number of people who could enter the isolation room at the same time (<4 people) because we found that there were more medical staff in the critical patient ward and unnecessary contact between medical staff increased the possibility of PPE and environmental contamination. The upper limit on the number of people allowed in was mainly based on the size of the room, and this restriction was only for routine work, does not include rescue and other special circumstances.
